# Evaluation of the effects of hyaluronic acid-carboxymethyl cellulose barrier on ovarian tumor progression

**DOI:** 10.1186/1757-2215-7-40

**Published:** 2014-04-16

**Authors:** Laetitia Picaud, Benoît Thibault, Eliane Mery, Monia Ouali, Alejandra Martinez, Jean-Pierre Delord, Bettina Couderc, Gwenael Ferron

**Affiliations:** 1 EA4553, Institut Claudius Regaud, 20-24 rue du pont St Pierre, 31052 Toulouse, France; 2 Surgery Department, Institut Claudius Regaud, 20-24 rue du pont St Pierre, 31052 Toulouse, France; 3 Biostatistic Unit, Institut Claudius Regaud, 20-24 rue du pont St Pierre, 31052 Toulouse, France; 4 Université de Toulouse, Université Paul Sabatier, 118, Route de Narbonne, F-31062 Toulouse, France

**Keywords:** Ovarian adenocarcinoma, Surgery, Anti-adhesion membranes, Hyaluronic acid

## Abstract

**Background:**

Hyaluronic acid is a prognostic factor in ovarian cancers. It is also a component of Hyaluronic Acid-Carboxymethyl Cellulose (HA-CMC) barrier, an anti-adhesion membrane widely used during abdominal surgeries in particular for ovarian carcinosis. 70% of patients who undergo ovarian surgery will relapse due to the persistence of cancer cells. This study’s objective was to determine the oncological risk from use of this material, in the presence of residual disease, despite the benefit gained by it decreasing post-surgical adhesions in order to provide an unambiguous assessment of its appropriateness for use in ovarian surgical management.

**Methods:**

We assessed the effects of HA-CMC barrier on the *in vitro* proliferation of human ovarian tumor cell lines (OVCAR-3, IGROV-1 and SKOV-3). We next evaluated, in vivo in nude mice, the capacity of this biomaterial to regulate the tumor progression of subcutaneous and intraperitoneal models of ovarian tumor xenografts.

**Results:**

We showed that HA-CMC barrier does not increase in vitro proliferation of ovarian cancer cell lines compared to control. In vivo, HA-CMC barrier presence with subcutaneous xenografts induced neither an increase in tumor volume nor cell proliferation (Ki67 and mitotic index). With the exception of an increased murine carcinosis score in peritoneum, the presence of HA-CMC barrier with intraperitoneal xenografts modified neither macro nor microscopic tumor growth. Finally, protein analysis of survival (Akt), proliferation (ERK) and adhesion (FAK) pathways highlighted no activation on the xenografts imputable to HA-CMC barrier.

**Conclusions:**

For the most part, our results support the lack of tumor progression activation due to HA-CMC barrier. We conclude that the benefits gained from using HA-CMC barrier membrane during ovarian cancer surgeries seem to outweigh the potential oncological risks.

## Background

Ovarian cancer is the leading cause of mortality among the gynecological diseases occurring in developed countries with 225,500 new cases and 140,200 estimated deaths worldwide [1,2]. This high rate of mortality is due in part because most patients presenting epithelial ovarian cancer have an advanced disease at the time of diagnosis [1]. After surgical resection, residual disease then represents the major pejorative predictive factor for survival [2]. For optimum results, surgery must be as complete as possible which generally means performing en-bloc resections of bowel, reproductive organs and genital tract, and peritonectomies.

The corollary of these “radical” surgeries is the induction of intra-abdominal adhesions that lead to the sticking together of two tissues normally moving freely past each other. As well as causing bowel obstruction and chronic pain, these adhesions can also induce heterogeneity of drug diffusion and compromise the delivery of adjuvant intraperitoneal chemotherapy [3]. Moreover, they can complicate any new surgical procedures, which in ovarian cancer disease are highly frequent due to the high rate of recurrence (70%). The place of secondary cytoreductive surgery is actually evaluated in several clinical trials with a complete resection as a gold standard. It is clear therefore that the reduction of adhesive disease has many immediate and delayed functional and therapeutic consequences in ovarian cancer bearing patients. An exhaustive review published by *Ward B.C* describes all adhesion prevention strategies including non-invasive surgical strategies, cellular strategies, pharmaceutical approaches, fluid and gel barriers, and lastly solid barriers including Hyaluronic Acid-Carboxymethyl Cellulose (HA-CMC) barrier [4].

HA-CMC barrier is an implantable and bioabsorbable synthetic membrane, composed of hyaluronan (also called hyaluronic acid or HA) and carboxymethylcellulose (CMC) which turns into a gel within 24 hours, before being absorbed from the surgical site within one week for elimination from the organism in less than 30 days. It became available in France in 2003 for indications of benign abdominal surgeries and since 2007, following Oikonomakis [5] and Kusonoki’s publications [6] for respectively colon and rectal cancers, is also used for carcinologic surgeries. Since then, many preclinical, animal and clinical works studying HA-CMC barrier in digestive cancers have been published. A meta-analysis performed by *Diamond MP* describes this material as « biocompatible, non-mutagenic, non-pyrogenic, non-irritating and non-toxic » [7]. However, some animal studies have given contradictory results revealing no effect of HA-CMC in colon tumor metastasis but a local increase in tumor growth [8], and in some cases, an increase in rate of abdominal metastasis [9].

The major component of HA-CMC barrier, HA, is a physiological component of extracellular matrix in association with collagen, proteoglycans (such as fibronectin, laminin and vitronectin) and elastin. Its main receptor is CD44 which is present at the surface of various normal or tumor cells. Another HA receptor, CD168, also called receptor hyaluronic acid mediated motility (RHAMM), has been described in CD44 knock-out mice [10]. The HA expression level is modified in the context of ovarian cancer and high levels are correlated with poor prognosis [11]. In histological retrospective studies, tumor stromal HA concentration has been linked with tumor aggressiveness, and stromal HA has been shown to be an independent prognostic factor for disease free and overall survival [12,13]. Moreover, high level of HA synthase 1 (HAS1), but not HAS2 and HAS3, is correlated with reduced overall survival in ovarian cancer [14]. Furthermore, CD44 expression level of ovarian cancer cells is inversely proportional to patient survival [15].

Many studies, mainly *in vitro*, have allowed us to define more precisely the implication of HA in ovarian cancer. As an example, HA from mesothelial cells, the major cell population in the peritoneum, is an adhesion factor for CD44-positive ovarian cancer cells [16]. These data have been confirmed by adhesion tests using hyaluronidase, anti-CD44 antibodies and CD44 targeted siRNA [17-19]. Mesothelial extracellular HA is also involved in ovarian carcinoma cell motility and dose-dependent chemotactism. Indeed, Boyden chamber tests highlighted than ovarian cancer cells preferentially migrate towards mesothelial cells as compared to control conditions, and that this effect is abolished using hyaluronidase [20]. Moreover, HA-CD44 interaction in SKOV-3 ovarian cancer cells can induce F-actin oriented polymerization using two signaling pathways involving Src or N-WASP [20-22].

Among non-gynaecological cancers, a retrospective study showed that HA-CMC barrier use has been associated with a 13% increased risk of abscess after colectomy [23]. On the contrary, HA-CMC barrier has not been showed to be associated with increased complications in thyroid surgery despite an uncertain anti-adhesive effect [24].

While some studies regarding HA-CMC barrier utilization in gynecological surgery have been published [25-29] none describe the potential effects of this biomaterial in ovarian metastatic process in animals. Of the mainly clinical studies, most are retrospective. Only one is prospective including 14 patients with HA-CMC barrier, and concern in half of the cases cervical and uterine cancers. Krill *et al.* evaluated the risk of postoperative complications related to HA-CMC utilization in ovarian cancers after cytoreductive surgery and showed that this biomaterial is not responsible for major complications but may be associated with increased risk of pelvic abscess [30]. However, they did not study the effects of HA-CMC on ovarian cancer progression and recurrence.

Our concern was to evaluate the potential effect of HA-CMC in a context where residual cancer cells were present. Indeed, after ovarian surgery, and because 70% of patients will relapse, it is clear that those cells could be affected by HA-CMC presence.

Accordingly, we decided to perform a preclinical study in order to evaluate HA-CMC barrier impact on ovarian tumor progression. *In vitro* experimentations were performed to analyze the effects of HA-CMC barrier on the proliferation of several ovarian cancer cell lines and on the activation of proliferative, survival and adhesion signaling pathways. *In vivo* mice xenograft models were used to evaluate the oncological risk of HA-CMC barrier utilization in comparison with expected benefits from adhesion prevention. Our ultimate aim was to provide a clear assessment of the appropriateness of using HA-CMC barrier in the surgical management of ovarian cancer. We demonstrated that *in vitro*, HA-CMC barrier did not increase tumor cell proliferation and that *in vivo*, except for anterior and lateral peritoneum implantation, HA-CMC barrier did not induce tumor growth.

## Methods

### Biomaterials

HA-CMC barrier membranes and absorbable material were granted from the surgical department of Claudius Regaud Institute (Toulouse France) without industrial founds.

### Cell culture

NIH ovarian adenocarcinoma cells (OVCAR-3 and SKOV-3) and HeLA cells were obtained from the American Type Culture Collection (ATCC® numbers HTB-161, HTB-77 and CCL-2). IGROV-1 ovarian adenocarcinoma cells were a gift from the Gustave Roussy Institute (Villejuif, France) and the REH lymphoblastic line was a gift from the hematology research unit of Toulouse University Hospital (France). All cells were cultured in RPMI medium supplemented with 10% fetal calf serum (FCS), penicillin/streptomycin (100 IU/mL/100 μg/mL) and 2 mM L-glutamine (Cambrex biosciences, Milan, Italy). Cell lines were routinely checked for mycoplasma.

### Cell proliferation

OVCAR-3, IGROV-1 and SKOV-3 cells were seeded in 6-well plates at a concentration of 2 × 10^5^ cells per well. Three different conditions were used: culture with RPMI alone or with 1 cm^2^ pieces of HA-CMC barrier or a control absorbable material. Cell proliferation kinetics was assessed by counting cells on a hemocytometer after 1, 2, 4 and 6 days of culture. Cell population doubling time during the exponential growth phase was calculated according to the following formula: T(hours) = t × [ln(2)/[ln(C1) ‒ ln(C0)]] in which t corresponds to the exponential growth phase in hours, C0 to the initial cell concentration at the beginning of this phase and C1 to the final cell concentration at the end of the phase.

### CD44 expression evaluation by flow cytometry

Each cell line (10^6^ OVCAR-3, IGROV-1, SKOV-3, HeLA or REH lymphoblast cells) was incubated during 45 minutes with a primary antibody directed against CD44 (1/100, [F10-44-2] (ab6124), Abcam, Paris, France), or against a control isotype (κ isotype Ctrl PE Mouse IgG1, BioLegend). Cells were then washed with PBS, centrifuged 5 minutes at 300 g, then incubated 45 minutes with an anti-mouse secondary antibody (1/50, BD Biosciences, Le pont de Claix, France). Cells were washed with PBS and the fluorescence was measured with a FACS Calibur cytometer and analyzed with Cell Quest Pro software.

### Animals

Four to five week-old female Swiss Nude athymic mice (Charles River laboratories, l’Abresle, France) were used after approval from Claudius Regaud Institute animal ethics committee (ICR-2012-017). They were housed according to the European Laboratory Animal Science Association standards. Mice experimentations began after one week of quarantine.

### HA-CMC barrier mouse absorption validation

A 2.25 cm^2^ (15 × 15 mm) piece of HA-CMC barrier was inserted into the abdomens of mice by median laparotomy after intraperitoneal general anesthesia (ketamine 50 mg/mL, xylazine 20 mg/mL and NaCl: 10 μg per gram of mouse). Mice were then sacrificed *via* cervical dislocation 1, 3 or 7 days after surgery in order to proceed to histological analysis.

### Subcutaneous (s.c.) xenografts

10^7^ SKOV-3 cells were injected subcutaneously into each of the right and left flanks of 16 mice. The day following this injection (D1), after cutaneous debridement with chlorhexidine, an incision was made under general anesthesia at the two injection sites and a 1 cm^2^ (10 × 10 mm) piece of HA-CMC barrier was alternatively inserted into one of the two sites. The other side was opened and then closed to reproduce the inflammation caused by the surgical procedure. The well-being of the mice was checked every 2 to 3 days. Tumor lengths (L) and widths (w) were also measured every two to three days and the tumor volume extrapolated using the formula: (π × length × width^2^)/6. Mice were sacrificed 21 days *via* cervical dislocation after the injection. Tumors were sampled then conditioned for histological analysis (formol fixation) or western blot (cryopreservation in liquid nitrogen).

### Intraperitoneal (i.p.) xenografts

2.5 × 10^7^ SKOV-3 cells were injected intraperitoneally into each of 22 mice. The day following the injection (D1), after cutaneous debridement with chlorhexidine, a laparotomy was performed under general anesthesia according to one of two procedures: (A) 11 mice underwent a “white” laparotomy (incision, abdominal opening and closure) and (B) 11 mice received an i.p. 2.25 cm^2^ (15 × 15 mm) piece of HA-CMC barrier. For both groups, parietal closure consisted in three separate absorbable material 3/0 sutures. The well-being of the mice was checked during 21 days and their weight was measured three times a week. The mice were sacrificed by cervical dislocation 21 days after the injection of cells. Autopsies were then performed by two double-blinded specialist surgeons in ovarian cancer surgery to evaluate the abdominal carcinosis index and tumors were removed for histological and western blot analysis.

### Histological analysis, Ki67 staining and mitotic index determination

Proliferative indices of tumor from s.c. or i.p. injections were assessed by immunohistochemical staining of paraffin embedded tumor sections for the proliferation marker Ki67 using mouse monoclonal MIB1 antibody. Ki67 positive nuclei were counted in random fields. The mitotic index was assessed by evaluating the number of cells in mitosis per high-power field (10 high-power fields per tumor).

### Western blot

Western blot was performed to evaluate *in vitro* protein expression in CD168 cell lines and to analyze *in vivo* the activation of signaling pathways within the xenografts. *In vitro,* proteins in OVCAR-3, IGROV-1, SKOV-3, HeLa and REH lymphoblastic cells were studied. *In vivo,* cryopreserved xenograft tumors were pooled according to protocol (subcutaneous *versus* intraperitoneal, with or without HA-CMC barrier). Cells and tumor samples were crushed then prepared with RIPA lysis buffer (Tris 1 M at pH 7.4, NaCl 150 mM, Triton, SDS 20%, MgCl_2_, NaF and protease inhibitors). The concentration of proteins was determined using the « Bradford Assay » (Bio-Rad) before their separation by SDS-PAGE in a 10% polyacrylamide gel and transfer onto a PVDF membrane (Amersham Hybond) previously activated with methanol. These membranes were saturated 45 minutes in TBS (50 mM Tris, 150 mM NaCl) / Tween 0.2% (TBST) supplemented with 5% milk and then incubated 90 minutes with a rabbit primary antibody against CD168 (1/1000, ab108339, Abcam) for cells lines, or Akt, p-Akt, p-ERK or FAK (1/1000, Cell Signaling Technology) for tumor samples. Membranes were washed three times with TBST and incubated 90 minutes with an anti-rabbit secondary antibody coupled with horseradish peroxidase (HRP) (1/2000, Cell Signaling Technology, St Quentin, France). Membranes were washed three times with TBST and twice with TBS before immunocomplexes were revealed with Enhanced Chemoluminescence (GE Healthcare) and visualized with a photon camera (Bio-Rad).

### Statistics

Quantitative variables were presented by median and range and qualitative data by frequency and percent. Group comparisons were made using the Wilcoxon signed rank test (matched) or Mann–Whitney test (independent) for non-parametric data and by the Student’s t test for parametric ones. For this entire study, statistical significance was reached for p < 0.05. *In vivo*, with a standard deviation of 0.1, to reach a statistical difference mean of 15% in terms of tumor proliferation with a statistical power of 80% and α risk of 5%, 22 mice per experimentation were necessary. All analyses were performed using STATA software (Version 12.0; Stata Corporation, College Station, TX, USA).

## Results

HA implication in ovarian tumor progression has already been described. The goal of this study was to determine whether HA-CMC barrier, being mainly composed of HA, was implicated in ovarian tumor progression and spread.

### OVCAR-3, IGROV-1 and SKOV-3 ovarian cancer cells express CD44 and CD168

**Figure 1:**
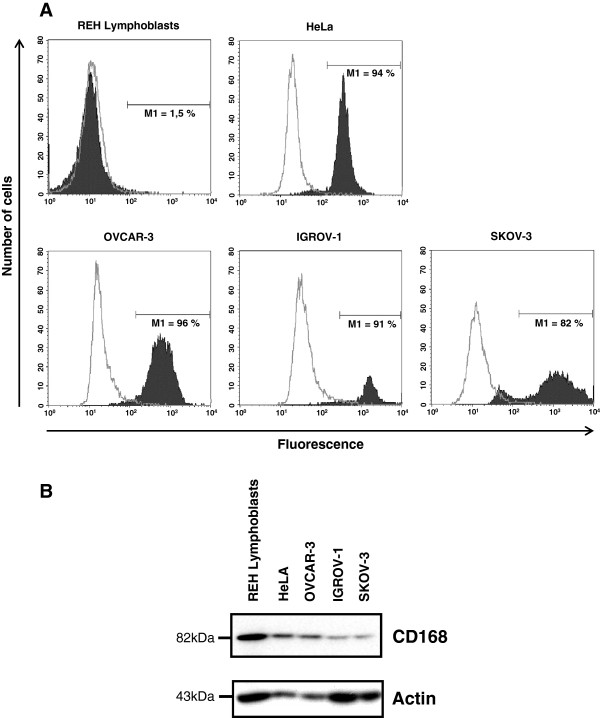
**CD44 and CD168 expression in ovarian cancer cell lines. (A)** The CD44 expression was measured by flow cytometry and analyzed with Cell Quest Pro software in ovarian cancer cell lines OVCAR-3, IGROV-1 and SKOV-3 as well as in REH lymphoblasts (negative control) and HeLa cells (positive controls). The auto fluorescence level was determined with an isotype control antibody (empty area). The CD44 expression was determined with an anti-CD44 antibody (full area) leading to the determination of the percentage of CD44 positive cells (M1). **(B)** The CD168 level was determined by western blot using a specific antibody in ovarian cancer cell lines OVCAR-3, IGROV-1 and SKOV-3 as well as in REH lymphoblasts and HeLa (positive controls). Immunocomplexes were visualized with a photon camera.

OVCAR-3, IGROV-1 and SKOV-3 cell lines differ in terms of their genetic alteration and chemosensitivity. In order to confirm the capacity of these cells to interact with HA-CMC barrier, we firstly studied their expression of HA receptors, CD44 and CD168 by respectively flow cytometry (Figure 1A) and western blotting (Figure 1B). As a negative control of CD44 expression, we used REH lymphoblast cells and as our positive control HeLa cells. REH lymphoblast cells and HeLa cells constituted our positive control for CD168 expression. All three ovarian cancer cell lines were shown to express both CD44 and CD168.

### HA-CMC barrier does not promote OVCAR-3, IGROV-1 or SKOV-3 ovarian cancer cell proliferation *in vitro*

**Figure 2:**
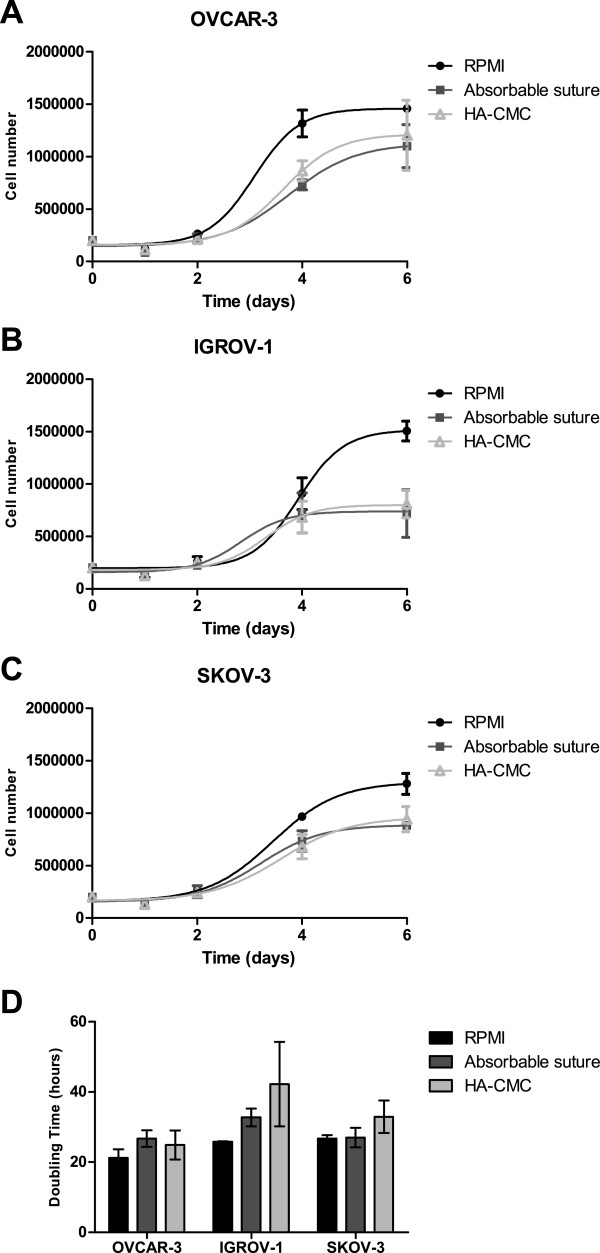
**Effects of HA-CMC barrier on ovarian cancer cell line proliferation *in vitro*. (A, B, C)** Ovarian cancer cell lines OVCAR-3 **(A)**, IGROV-1 **(B)** and SKOV-3 **(C)** were cultured in the presence of control medium (RPMI), 1 cm^2^ of control absorbable material, or 1 cm^2^ of HA-CMC barrier. After 1, 2, 4 and 6 days of culture, cell number was counted with a hemocytometer. (Mean number of cells +/− SEM, n = 3). **(D)** Doubling times were calculated from previous proliferation curves according to the following formula: doubling time (hours) = t x [ln (2) / [ln (C1) – ln (C0)]] with t corresponding to the duration of the exponential phase, C0 to the initial cell concentration and C1 to the final cell concentration (between the beginning and the end of the exponential phase). (Mean doubling time +/− SEM, n = 3).

To evaluate the effect of HA-CMC barrier on ovarian cancer cell proliferation, OVCAR-3, IGROV-1 and SKOV-3 cells were seeded in medium alone or with 1 cm^2^ pieces of HA-CMC barrier or a control absorbable material which is a biomaterial used in abdominal surgery that, as an added exogenous material induces inflammation *in vivo. In vitro,* absorbable material was used as a control for biomaterial presence. We used an hemocytometer to count the cells for each condition 1, 2, 4 and 6 days after seeding (Figures 2A, B and C) and calculated the doubling time for each cell line during the exponential proliferative phase between the 2nd and 4th day (Figure 2D).

Comparison to the control condition (RPMI medium alone) revealed a lack of any cell proliferation induced by either HA-CMC barrier or absorbable material (Figure 2A, B and C). Indeed, less cells could be defined at day 6 in the HA-CMC barrier group even though there was no statistical difference regarding cell line doubling times between the 2 conditions.

Absorbable material absorption was longer than that for HA-CMC barrier which, considering the potential to lead to chronic inflammation in mice, we decided to exclude this biomaterial from *in vivo* experiments.

### Absorption of HA-CMC barrier is similar between mice and women

**Figure 3:**
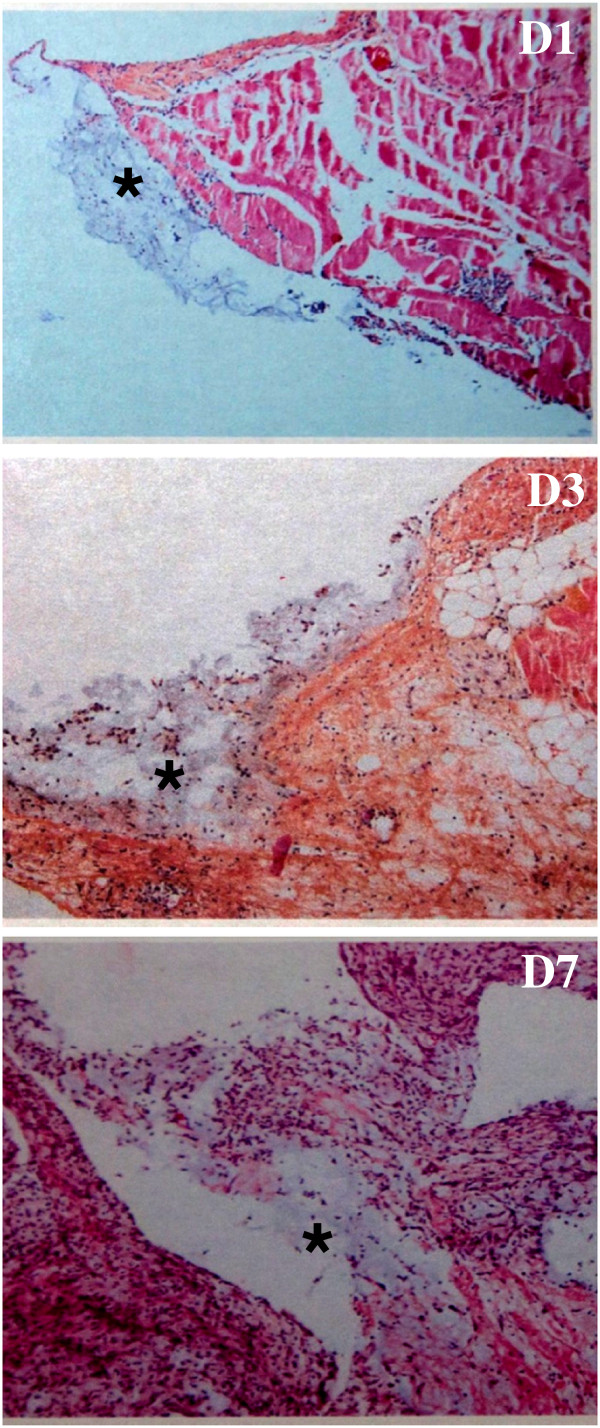
**Evolution of the HA-CMC barrier absorption in nude immunosuppressed mice.** After general anesthesia and cutaneous debridement, a 15 x 15 mm piece of HA-CMC barrier was placed in mice abdomens. 1 (D1), 3 (D3) and 7 (D7) days after surgery, mice were sacrificed and abdominal walls were analyzed by histology (hematein-eosin coloration). HA-CMC barrier pieces or residues were marked with a star (*).

In order to evaluate whether the HA-CMC barrier absorption in mice is representative of that occurring in humans, we performed a laparotomy and place HA-CMC barrier pieces into the abdomen of mice. The mice were then sacrificed 1, 3 or 7 days after surgery before histological analysis of the surgical site was performed in order to observe the biomaterial presence (Figure 3). After one day, HA-CMC barrier was present as an amorphous and cell-free material in association with numerous neutrophilic polynuclear cells (acute inflammatory reaction). After 3 days, HA-CMC barrier persisted as subperitoneal sediment and the acute inflammatory reaction was associated with mesothelial hyperplasia. At day 7, HA-CMC barrier remaining under the peritoneum had been mostly absorbed, and an inflammatory phase going into the proliferative phase of wound healing was evident by the presence of fibroblasts and macrophages at the peritoneal surface.

### Subcutaneous (s.c.) xenografts

**Figure 4:**
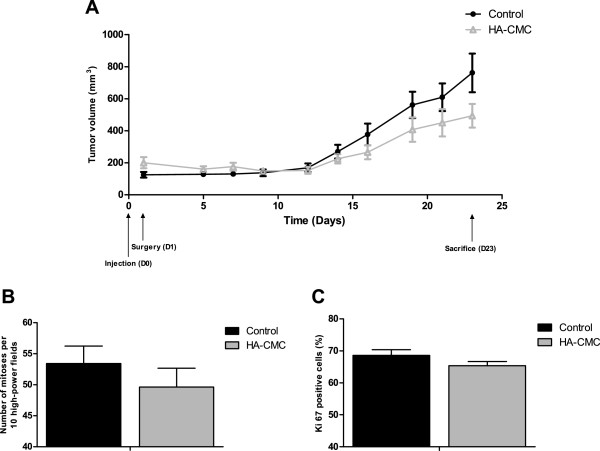
**Impact of HA-CMC barrier on ovarian s.c. tumor xenografts. (A)** SKOV-3 cells were injected subcutaneously into the flanks of immunosuppressed mice at day 0 (D0). At day 1 (D1), a 1 cm^2^ piece of HA-CMC barrier was inserted into one flank of each mouse (HA-CMC barrier), the other flank being simply opened and closed again (control). Tumor volume was extrapolated from the measurement of tumor length and width every 2 or 3 days. (Mean tumor volume +/− SEM) 23 days after the injection, mice were sacrificed and tumors were sampled for histological analysis. **(B)** Mitotic index was determined by counting the number of mitoses per 10 high-power fields. **(C)** Ki67 positive cells (%) were determined by Ki67 marking.

To determine the potential role of HA-CMC barrier in ovarian tumor growth, we established SKOV-3 cell s.c. xenografts in mice in both flanks. The day following this injection, we made an incision at the two injection sites and placed a 1 cm^2^ piece of HA-CMC barrier, proportionally similar to the mean surface area during human ovarian cancer surgery, randomly into one of the two flanks, the other side simply being closed again to reproduce the inflammation caused by the surgical procedure. Tumor volume, as extrapolated from the measurement of tumor length and width every 2 or 3 days (Figure 4A), showed no increase in the presence of HA-CMC barrier by comparison with control. Median tumor volume in fact showed a greater variation between days 12 and 21 in the control group than in the HA-CMC barrier group (p = 0.0288). Histological analysis of all tumor samples showed that by comparison with the control group, the HA-CMC barrier group had neither an increased mitotic index (Figure 4B) nor Ki67 marking (Figure 4C), highlighting that this biomaterial had no effect on the growth of the s.c. ovarian tumor xenograft.

### Intraperitoneal (i.p.) xenografts

In order to study the possible effect of HA-CMC barrier on ovarian tumor peritoneal dissemination, we then established i.p. SKOV-3 cell xenografts in mice. The day following the injection, mice underwent a “white” laparotomy (incision, abdominal opening and closure) or a laparotomy with an i.p. implementation of a 2.25 cm^2^ piece of HA-CMC barrier. These mice were sacrificed by cervical dislocation 21 days after this injection before two surgeons, specialists in ovarian cancer surgery, performed an autopsy to evaluate murine carcinosis index (MCI) per organ (Table 1). We modified a previously published endoscopic murine score of carcinosis [31], that made it applicable to macroscopic analysis.

$$\begin{array}{l} \mathrm{Total}\;\mathrm{MCI}\;\mathrm{score}=\mathrm{nodule}\;\mathrm{maximal}\;\mathrm{diameter}\;\mathrm{score}\\ \;\times \mathrm{nodule}\;\mathrm{number}\;\mathrm{score} \end{array}$$

**Table 1:** Determination of the murine carcinosis index (MCI)

**Maximal nodule diameter**	**No visible disease**		**< 0.5 mm**	**0.5 to < 2 mm**		**≥ 2 mm**
**Diameter score**	0		1	5		10
**Nodule number**	1-5	6-10	11-15	16-20	21-25	>25
**Number score**	1	2	3	4	5	6

The MCI per organ (diaphragm, liver, peritoneum, digestive tract) is determined in mice by the following formula: total score = nodule maximal diameter score × nodule number score. The diameter score corresponds to the maximal nodule diameter (from non-visible nodules to nodules up to 2 mm diameter or over). The number score corresponds to the number of nodules in the organ (from 1-5 nodules up to 25 nodules or over).

**Figure 5:**
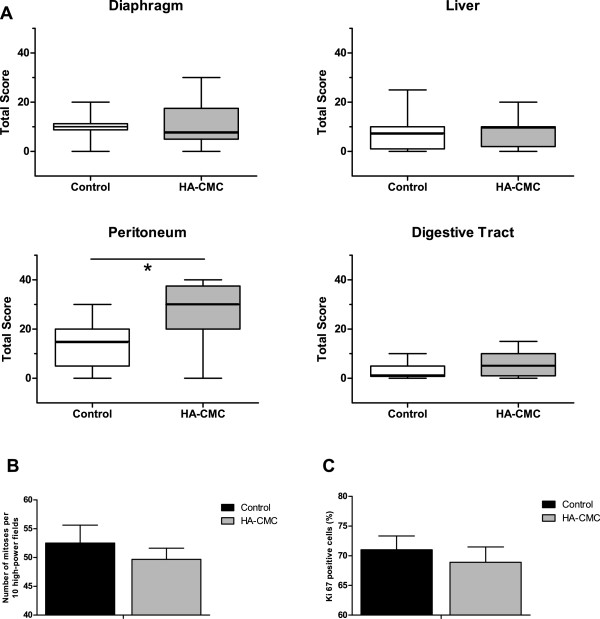
**Impact of HA-CMC barrier on ovarian i.p. tumors xenograft. (A)** SKOV-3 cells were injected intraperitoneally into the abdomen of immunosuppressed mice. The day following the injection, half of the mice received a “white” laparotomy (control) and the other half underwent a laparotomy with the insertion of a 2.25 cm^2^ piece of HA-CMC barrier (HA-CMC barrier). 21 days after the injection, mice were sacrificed and a macroscopic analysis was performed in order to evaluate the median peritoneal carcinosis index for each of diaphragm, liver, peritoneum and digestive tract. (Median +/− Extreme values, *p < 0.05) Tumors were sampled for histological analysis. **(B)** Mitotic index was determined by counting the number of mitoses per 10 high-power fields. **(C)** Ki67 positive cells (%) were determined by Ki67 marking.

Our results showed that HA-CMC barrier induced no increase in MCI in the diaphragm, liver or digestive tract by comparison with the control group. However, HA-CMC barrier was responsible for a significantly higher MCI score (30 compared to 15, p = 0.0349) in the anterior and lateral peritoneum (excluding the diaphragmatic cupola) compared to the control group (Figure 5A).

Histological analysis revealed no statistical difference between HA-CMC barrier and control groups with regards mitotic index (Figure 5B) or percentage of Ki67 positive cells (Figure 5C).

### HA-CMC barrier does not activate MAP kinase, Akt or FAK pathways in i.p. or s.c. ovarian tumor xenografts

**Figure 6:**
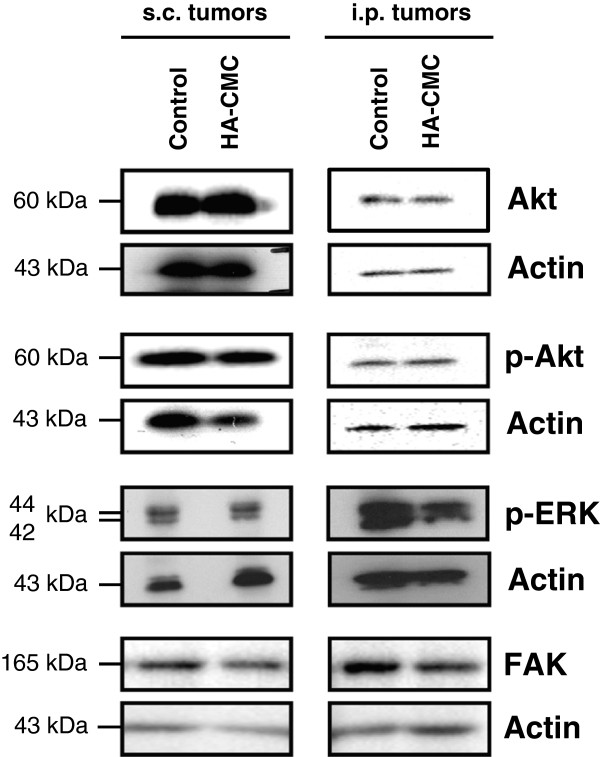
**Effects of HA-CMC barrier on proliferation, survival and adhesion signaling pathwasy in ovarian tumor xenografts.** s.c. and i.p. tumors were sampled the day the mice were sacrificed and were grouped together according to experimental condition (control or HA-CMC barrier). The Akt, phospho-Akt (p-Akt), phospho-ERK (p-ERK) and FAK levels were determined by western blot using a specific antibody. Immunocomplexes were visualized with a photon camera.

With regards the participation of peritumoral stroma and microenvironment cells, except for the increase in MCI in the anterior and lateral peritoneum, we observed no phenotypical modifications concerning proliferation, growth and tumor dissemination caused by HA-CMC barrier. To investigate potential molecular effects of this biomaterial on ovarian tumor cells, we assessed the activation of cell signaling pathways implicated in cell survival (Akt and phospho-Akt), proliferation (phospho-ERK, the terminal kinase of the MAP-kinase pathway), and adhesion (FAK). Our results show that HA-CMC barrier did not modify the level of Akt or FAK and did not induce phosphorylation of Akt or ERK when compared to the control group (Figure 6). Therefore, on the basis of studied proteins, it appeared that HA-CMC barrier did not induce the activation of cell survival, proliferation or adhesion signaling pathways in either s.c. or i.p. ovarian tumor xenografts.

## Discussion

Complete cytoreductive surgery for advanced ovarian cancer can induce extended abdominal adhesions. These adhesions may prevent optimal intraperitoneal treatment delivery and can cause bowel obstruction and pain. The need to prevent such adhesions during abdominal surgery is a unanimously agreed objective. Among the solid barriers placed to decrease adhesive disease, HA-CMC barrier is one of the most widely used, and yet its essential component HA is a prognostic factor of survival in ovarian cancer bearing patients. Despite our in-depth study of the literature, we found no conclusive data regarding the oncological risk of using HA-CMC barrier in these patients.

Our results show that HA-CMC barrier does not activate the proliferation of the ovarian tumor cells OVCAR-3, IGROV-1 or SKOV-3 *in vitro*. Moreover, it cannot be excluded that the biomaterials may have actually acted as a physical constraint of cell proliferation. Indeed, whether the biomaterial used was HA-CMC barrier or an absorbable material, cell proliferation was less active than without the biomaterial. Particularly with IGROV-1 cells, HA-CMC seemed to present an anti-proliferative effect. Observing the same number of IGROV-1 cells in each condition at day 1 (Figure 2B and D), we did not conclude on an anti-adhesive effect of HA-CMC barrier on IGROV-1 cells. Considering that *in vitro* experiments exclude microenvironment participation, we studied *in vivo* the potential implication of HA-CMC barrier in ovarian tumor progression in mice.

Nearly all the results obtained *in vivo,* whether they concerned subcutaneous tumor cell injections (tumor volume, mitotic index or Ki67 percentage) or intraperitoneal xenografts (mean weight of mice, median carcinosis score of the diaphragm, liver and intestine, mitotic index or Ki67 percentage), we observed no proliferation increase associated with the HA-CMC barrier. Our findings corroborate other studies concerning non gynecological cancer in which HA-CMC barrier was described to protect trocar sites from gall bladder metastasis and abdominal wall implantation [32] while not decreasing overall survival for colon and rectal cancers [33].

However, our study does highlight that an i.p. HA-CMC barrier implantation can induce a statistically significant increase in the peritoneum carcinosis score (excluding hemi diaphragm). While this was the only detected pejorative impact of HA-CMC barrier, Hubbard et al. did publish similar findings for KM12-L4 colon cancer xenografts in mice, observing a local increase in tumor volume in the sidewall of the HA-CMC barrier and absorbable material groups. This increase was thought to be due to local inflammation induced by the presence of an exogenous biomaterial [8].

This result does warrant some concern, but such tumor growth was isolated in our study. Human peritoneal carcinosis extension and resecability evaluation are respectively performed with Sugarbaker and Fagotti scores but there is no equivalent for murine carcinosis experimentation. We decided to elaborate our own scoring system, which is applicable macroscopically without any endoscopic or informatics systems contrary to Matsuzaki published score [31]. There were some differences between the two double-blinded evaluation but the mean difference concerning the peritoneal carcinosis score, which constitutes the only localization apparently impacted by HA-CMC barrier, was only 6.83%.

Since we were unable to highlight a clear implication of HA-CMC barrier in ovarian tumor progression using macroscopic parameters, we focused on a potential effect of this biomaterial on molecular activation. To this end, we studied potential alteration in the expression of proteins involved in cell survival (total Akt and phospho-Akt), cell proliferation (phospho-ERK) and adhesion (FAK). These signaling pathways have been described in the literature as being activated following AH-CD44 interactions (PI3K-Akt [34], ERK [35] or FAK [36]). However we observed no activation of these different pathways in the HA-CMC barrier group of tumors by comparison with control tumors thus confirming the lack of effect of this biomaterial present on ovarian tumor behavior.

HA-CMC barrier-derived HA must therefore be differentiated from extracellular endogenous HA, the expression of which favors the evolution of ovarian cancer carcinosis. One of the possible explanations for the safety of HA-CMC barrier-derived HA could be the difference in its molecular weight compared to endogenous HA; high weight HA (4000 MDa) is anti-angiogenic and non-immune response-inducing and low weight HA (10 MDa) is pro-inflammatory, pro-angiogenic and immune response-inducing [37]. Another explanation could be that HA-CMC barrier HA shows less affinity for CD44-expressing tumor cells. To test such a hypothesis, it would be interesting to determine the phosphorylation status of CD44 receptors in response to HA-CMC barrier placement *in vitro*. Endogenous HA effects are likely the consequence of several interactions, not only with tumor cells but also with the tumor microenvironment. Moreover, they may also depend on their combined activation of surface receptors with other extracellular matrix components such as vitronectin or fibronectin.

We performed our *in vivo* experiments on nude mice models. These animals presented a lack of mature T cells that could modify the response of ovarian tumors to HA-CMC barrier. However, we focused on human tumor cells behavior in nude mice where macrophages, neutrophils and NK cells were present and responsible for a local inflammation. Indeed, we showed an acute inflammatory reaction 3 and 7 days after HA-CMC barrier injection in nude mice. We concluded that the absence of T cells did not alter the inflammation process in our model. Moreover, the describing functions of all T cells subtypes (CD8+ T cells, T helpers cells, regulatory T cells and γδ T lymphocytes) in ovarian cancer progression showed opposite roles [38]. So, we decided to work with immunodeficient mice allowing the utilization of human tumor cells compared to immunocompetent mice requiring murine cells.

Our results are worrisome because of MCI increase in anterior and lateral peritoneum of HA-CMC barrier treated mice. However, the clinical benefit of HA-CMC barrier utilization is to our mind clearly established. First, ovarian cancer bearing patients need to undergo multiple surgeries that lead to the development of adherences which constitute a complication risk factor. Secondly, intraperitoneal chemotherapy delivery cannot be performed because of adherences which induce heterogeneity of chemotherapy diffusion [3]. Because of benefit/risk balance, we decided to use this biomaterial for our patients keeping in mind a clinical prospective study remains necessary in order to clearly establish the harmlessness of HA-CMC barrier.

## Conclusion

In this study, we have shown that HA-CMC barrier does not induce the proliferation of ovarian tumor cell lines OVCAR-3, IGROV-1 and SKOV-3 *in vitro*. We also highlighted that *in vivo*, with the exception of anterior and lateral peritoneum implantation, it does not regulate ovarian tumor growth or dissemination of s.c. or i.p. xenografts in mice. Moreover, HA-CMC barrier did not activate microscopic cell proliferation (mitotic index and Ki67 marking) or survival, proliferation and adhesion signaling pathways. Only the median peritoneal score (excluding hemi diaphragm) was increased by HA-CMC barrier use. This effect was probably linked to the inflammatory response due to exogenous biomaterial. Altogether we conclude that HA-CMC barriers seem to provide more benefits than potential oncological risks even if we still cannot prove it does not increase locally the growth of potentially residual tumor cells remaining after surgery. The only way to confirm HA-CMC barriers safety is to lead a prospective clinical study to assess neither recurrence rate increase nor disease-free survival decrease for extended ovarian cancer suffering patients.

## Abbreviations

ATCC: American type culture collection; CMC: Carboxymethylcellulose; FCS: Fetal calf serum; HA: Hyaluronic acid; HRP: Horseradish peroxidase; i.p.: Intraperitoneal; MCI: Murine carcinosis index; RHAMM: Receptor hyaluronic acid mediated motility; s.c.: Subcutaneous.

## Competing interests

The authors declare that they have no competing interest.

## Authors’ contributions

LM and BT carried out the research and prepared the original manuscript. EM carried out the immunohistochemistry experiments. MO was in charge of the statistical analysis. AM and GF carried out the mouse observation for murine carcinosis score obtention. BT, JPD, BC and GF supervised the research and directed the project. All authors approved the final manuscript.
